# High versus standard doses interferon-alpha in the treatment of naïve chronic hepatitis C patients in Taiwan: a 10-year cohort study

**DOI:** 10.1186/1471-2334-5-27

**Published:** 2005-04-12

**Authors:** Ming-Lung Yu, Chia-Yen Dai, Shinn-Cherng Chen, Li-Po Lee, Ming-Yen Hsieh, Zu-Yau Lin, Ming-Yuh Hsieh, Liang-Yen Wang, Jung-Fa Tsai, Wen-Yu Chang, Wan-Long Chuang

**Affiliations:** 1 Hepatobiliary Division, Department of Internal Medicine, Kaohsiung Medical University Hospital, No. 100, Tzyou 1st Rd, Kaohsiung 807, Kaohsiung, Taiwan; 2 Department of Occupational Medicine, Kaohsiung Municipal HsiaoKang Hospital, No. 482, Shan-Ming Rd, Kaohsiung 812, Kaohsiung, Taiwan

## Abstract

**Background:**

Interferon-alpha monotherapy is effective in less than one-third patients with chronic hepatitis C. The dose-effect, tolerability and durability of interferon-alpha treatment and its long-term effect on the prevention of cirrhosis and hepatocellular carcinoma in naïve Taiwanese patients with chronic hepatitis C have not been well investigated. We conducted the present cohort study treated with high and standard interferon-alpha to illustrate the issues.

**Methods:**

We performed a long-term virologic and histological follow-up of 214 chronic hepatitis C patients treated with interferon-alpha, 3 million units (3-MU, n = 80) or 6-MU (n = 134) thrice weekly for 24 weeks, in Taiwan between 1992 and 2001.

**Results:**

There was no difference in the incidence of discontinuation between 3-MU and 6-MU groups (4/80, 5.0% versus 10/134. 7.5%). The 6-MU group had similar incidence of adverse events with the 3-MU group, except that 6-MU group had significantly higher incidence of psychological manifestations, mainly presented as irritability. The rates of sustained virological response (SVR) were significantly higher in 6-MU regimen (37.1%) than in 3-MU regimen (23.7%, p < 0.05) in per protocol analysis. Based on multivariate analysis, baseline viral load was strongly associated with SVR, followed by hepatitis C virus genotype, interferon-alpha regimen, and liver fibrosis. A histological improvement in necroinflammatory activity, but not in fibrosis was observed in the follow-up biopsy performed 0.5–5.5 years (mean: 1.9 years, n = 51) after end-of-treatment. Among patients without SVR, there was more activity improvement in 6-MU group. The durability of SVR was 100% (18/18) and 97.8% (45/46) for 3-MU and 6-MU group, respectively, in a mean follow-up period of 6.81 years (5.25–9.18 years). For 163 baseline non-cirrhotic patients, 9 of 84 (10.7%) non-responders and 3 of 79 (3.8%) sustained responders progressed to cirrhosis during a mean follow-up period of 5.52 and 5.74 years, respectively (p = 0.067, Kaplan-Meier survival analysis, log-rank test). For all 200 patients, hepatocellular carcinoma was detected in 12 of 113 (10.6%) non-responders and one of 87 (1.1%) sustained responders during a mean follow-up period of 5.67 and 5.73 years, respectively (p < 0.01, Kaplan-Meier survival analysis, log-rank test).

**Conclusion:**

We confirm the dose effect of interferon-alpha in chronic hepatitis C. Six-MU regimen had better efficacy than 3-MU regimen in virologic and histological responses. Both regimens had good tolerability and durability in Taiwan. Sustained response could reduce the incidence of cirrhotic change and hepatocarcinogenesis.

## Background

Hepatitis C virus (HCV) is the major etiologic agent in parenterally transmitted non-A non-B hepatitis and frequently causes persistent infection leading to chronic liver disease and primary hepatocellular carcinoma (HCC)[1,2]. Interferon-alpha (IFN-α) was the first approved therapy in the 1980s but resulted in a sustained virological response in only 8–20% of chronic hepatitis C patients treated with a standard regimen of IFN-α monotherapy, 3 million units (MU) thrice weekly for 24 weeks [3-8]. A number of factors have been considered in terms of their potential to predict the response to IFN-α therapy. These include infection with non-genotype 1b, lower levels of viremia and the absence of cirrhosis, which have been currently reported to be associated with a better response[2,9,10]. Several studies in Japan and Western countries have shown that higher dose (5–10 MU) of IFN could improve the efficacy on chronic hepatitis C[5,9,11]. Reviewing the Medline, however, only one limited study on the efficacy of IFN therapy for naïve patients of chronic hepatitis C has been reported before 1997 in Taiwan[4]. Furthermore, the dose effect, tolerability and durability of high-dose IFN therapy for naïve Taiwanese chronic hepatitis C patients have never been reported.

Since 1992, we have treated a number of Taiwanese patients with chronic hepatitis C with a standard IFN regimen, 3 MU thrice weekly for 24 weeks, and since 1995, with 6 MU thrice weekly for 24 weeks. We have followed these patients closely for 5–10 years, and we report herein the end-of-treatment and sustained response, and results of long-term follow-up to both regimens, as well as the dose effect, tolerability and durability of high-dose IFN therapy.

## Methods

### Patients

Two hundred and fourteen naïve chronic hepatitis C patients were enrolled in the study. All were positive for HCV antibodies (second-generation, enzyme-linked immunosorbent assay, Abbott, North Chicago, IL) and serum HCV RNA for at least 6 months. Patients with a concurrent hepatitis B virus, alcohol abuse (≧80 mL ethanol per day), overt hepatic failure, a current or past history of psychiatric condition, pregnancy, or with evidence of hepatocellular carcinoma were excluded. Two pathologists assessed all biopsy results, which were taken before IFN treatment, without knowledge of patients' clinical or laboratory data. Disease activity grade and fibrosis stage were quantitatively scored according to the histological activity index described by Knodell et al[12] Cirrhosis was diagnosed histologically in the presence of a stage 4 of fibrosis or in the absence of a liver biopsy, by a compatible ultrasonographic and clinical picture. A follow-up liver biopsy was performed in 51 patients. The present study was approved by the ethics committee of Kaohsiung Medical University Hospital. After they had given their informed consent, all patients were treated with recombinant IFN-α 2a (n = 40), IFN-α 2b (n = 102) or lymphoblastoid IFN-α n1 (n = 72), given intramuscularly, in a dose of 3-or 6-MU thrice weekly for 24 weeks. Group allocation was chronological and consecutive rather than randomized, because, before 1994, all patients were given a 3-MU and then a 6-MU IFN-α regimen. Fourteen patients (4 in 3-MU group, 5.0% and 10 in 6-MU group, 7.5%), who received IFN-α therapy less than 12 weeks, were excluded from the present study (Table 1). The presence of HCV RNA in the serum was assessed every three months for 12 months, and then every 12 months. End-of-treatment virological responder (ETVR) was defined as patients showing clearance of HCV RNA at the end of treatment. Others were defined as non-ETVR. Sustained virological responder (SVR) was defined as patients showing clearance of HCV RNA by at the end-of-treatment and 6 months after end-of-treatment. The others were classified as non-SVR. Histological improvement and worsening was defined as a ≧2-point decrease and increase in the total necroinflammatory scores between paired biopsies, respectively.

**Table 1:** Incidence of discontinuation and adverse events

	3-MU group, No. (%)	6-MU group, No. (%)	P value
Patient number	80	134	
Discontinuation	4 (5.0)	10 (7.5)	NS
Adverse event	3 (3.8)	6 (4.5)	NS
Insufficient response	1 (1.3)	1 (0.7)	NS
Laboratory abnormality	0 (0)	1 (0.7)	NS
Economic problem	0 (0)	2 (1.5)	NS
			
Adverse event			
Flu-like symptoms†	49 (61.3)	85 (63.4)	NS
Gastrointestinal manifestations‡	19 (23.8)	35 (26.1)	NS
Psychological manifestations§	34 (41.0)	74 (55.2)	0.028
Alopecia	13 (16.3)	30 (22.4)	NS
Dermatological manifestations¶	11 (13.8)	19 (14.2)	NS

Note: 3-MU group: interferon-α 3 million units thrice weekly for 24 weeks.

6-MU group: interferon-α 6 million units thrice weekly for 24 weeks.

NS: not significant.

† Including fatigue, headache, pyrexia, myalgia, and rigors.

‡ Including nausea, vomiting, anorexia, and diarrhea.

§Including irritability, depression, and insomnia.

¶Including dermatitis, and pruritus.

### Detection/quantification of serum HCV RNA and genotyping

Detection of serum HCV RNA was performed using a standardized automated qualitative reverse transcription polymerase chain reaction assay (RT-PCR, COBAS AMPLICOR Hepatitis C Virus Test, version 2.0; Roche, Branchburg, NJ, USA). The detection limit was 50 IU/mL. HCV genotypes 1a, 1b, 2a, 2b and 3a were determined by amplification of the core region using genotype-specific primers described by Okamoto et al[13]. Serum HCV RNA levels were measured by using the branched DNA assay (Quantiplex HCV RNA 2.0, Bayer, Emeryville, CA), performed strictly in accordance with the manufacturer's instructions. The quantification range was 0.2 to 120 million equivalents (Meq) of HCV RNA per ml.

### Statistical analyses

Frequency was compared between groups using the chi-square test with Yate's correction or Fisher's exact test. Group means were compared using the Student *t*-test. Serum HCV RNA levels were expressed as the mean ± standard deviation after logarithmic transformation of original values. Stepwise logistic regression was used to analyze factors associated with IFN-α response. Comparisons of paired liver histology were carried out with two-sample Wilcoxon's signed rank test. Preventive effects of antiviral therapy on progression of chronic HCV infection to liver cirrhosis (LC) and HCC were analyzed by using Kaplan-Meier survival analysis.

## Results

### Adverse effect

There was no difference of incidence of discontinuation of treatment between 3-MU and 6-MU groups (Table 1). The incidence of adverse events was similar between two groups in the presentation of flu-like symptoms, gastrointestinal and dermatological manifestations, and alopecia. However, patients in 6-MU group had significantly higher rate of psychological manifestation than those in 3-MU group did (74/134, 55.2% versus 34/80, 41.0%, p = 0.028, Table 1).

### SVR and factors predicting SVR

Seventy-six patients received 3-MU IFN-α and 124 received 6-MU, thrice weekly for 24 weeks. All were followed-up for at least 6 months after end of treatment. Baseline clinical and laboratory data were not different in the two groups (Table 2). At the end of IFN-α therapy, 36 patients (47.4%) in 3-MU group and 103 (83.1%) had ETVR, the difference being highly significant (P < 0.0001). Of those with ETVR, 50% (18/36) in 3-MU group and 55.3% (57/103) in 6-MU group had reappearance of serum HCV RNA within 6 months after end-of-treatment. The difference was not significant. The SVR was significantly higher in 6-MU group (37.1%, 46/124) than in 3-MU group (23.7%, 18/76, P < 0.05).

**Table 2:** Baseline demographic and clinical features of 200 chronic hepatitis C patients with interferon-alpha therapy

	Total	3-MU group† No. (%)	6-MU group‡ No. (%)
Patient Number	200	76	124
Gender			
Male	103	34(44.7)	69(55.6)
Female	97	42(55.3)	55(44.4)
Age (year)		47.3 ± 10.4	45.3 ± 11.7
History of transfusion			
No	141	56(73.7)	85(68.5)
Yes	59	20(26.3)	39(31.5)
Liver histopathology			
Total score of necroinflammatory activity		4.48 ± 2.56	4.23 ± 2.46
Fibrosis score			
F3–4	70	30(39.5)	40(32.3)
F0–2	130	46(60.5)	84(67.7)
Pretreatment ALT value (U/L)		117.4 ± 102.8	104.1 ± 95.4
Pretreatment HCV RNA level (log equivalent/mL)		5.97 ± 0.59	6.01 ± 0.68
HCV genotype			
1b	80	27(35.5)	53(42.7)
Non-1b	120	49(64.5)	71(57.3)
Interferon preparation			
Recombinant IFN-α-2a	37	10(13.2)	27(21.8)
Recombinant IFN-α-2b	95	36(47.4)	59(47.6)
Lymphoblastoid IFN-α-n1	68	30(39.5)	38(30.6)

† 3-MU group: interferon-α 3 million units thrice weekly for 24 weeks.

‡ 6-MU group: interferon-α 6 million units thrice weekly for 24 weeks.

In univariate analysis, SVR was not related gender, age, history of transfusion, the necroinflammatory activity of liver histopathology, or preparations of IFN-α (Table 3). A significant positive association for SVR was found for pretreatment alanine aminotransferase value and IFN-α dose, and a negative association with severe fibrosis (score 3 or 4), infected with HCV genotype 1b and pretreatment serum levels of HCV RNA. Based on multivariate logistic regression analysis, pretreatment HCV RNA level was strongly associated with SVR, followed by infected HCV genotype, IFN-α regimen, and severity of liver fibrosis (table 4).

**Table 3:** Factors associated with sustained viral response to interferon-alpha therapy

	Total No.	Non-SVR† No. (%)	SVR† No. (%)	P value
Patient Number	200	136(68.0)	64(32.0)	
Gender				NS‡
Male	103	69(70.0)	34(33.0)	
Female	97	67(69.1)	30(30.9)	
Age (year)		47.6 ± 10.0	44.2 ± 13.5	NS
History of transfusion				NS
No	141	97(68.8)	44(31.2)	
Yes	59	39(66.1)	20(33.9)	
Liver histopathology				
Total score of necroinflammatory activity		4.10 ± 2.59	4.21 ± 2.31	NS
Fibrosis score				<0.01
F3–4	70	57(81.4)	13(18.6)	
F0–2	130	79(60.8)	51(39.2)	
Pretreatment ALT value (IU/L)		87.8 ± 86.5	143.5 ± 153.5	<0.01
Pretreatment serum level of HCV RNA§		6.17 ± 0.61	5.62 ± 0.56	<0.0001
HCV genotype				
1b	80	63(78.8)	17(21.3)	<0.01
Non-1b	120	73(60.8)	47(39.2)	
Interferon preparation				NS
Recombinant IFN-α-2a	37	29(78.4)	8(21.6)	
Recombinant IFN-α-2b	95	62(65.3)	33(34.7)	
Lymphoblastoid IFN-α-n1	68	45(66.2)	23(33.8)	
Interferon regimen				<0.05
3-MU group¶	76	58(76.3)	18(23.7)	
6-MU group¶	124	78(62.1)	46(37.1)	

† SVR: sustained virological response.

‡ NS: not significant.

§Presented as log (equivalent/mL).

¶ 3-MU group: interferon-α 3 million units thrice weekly for 24 weeks; 6-MU group: interferon-α 6 million units thrice weekly for 24 weeks.

**Table 4:** Multivariate logistic regression analysis of factors associated with sustained viral response to interferon-alpha therapy in chronic hepatitis C patients

Variable†		Odds ratio	95% CI‡
Pretreatment HCV RNA level§	Per 1 log increase	0.179	0.096–0.336
HCV genotype	1b = 1; non-1b = 0	0.324	0.151–0.695
IFN-α regimen	6-MU = 1; 3-MU = 0¶	2.310	1.070–4.990
Stage of fibrosis	F3–4 = 1; F0–2 = 0	0.408	0.104–0.892

† Factors included, age, sex, history of transfusion, pretreatment serum level of alanine aminotransferase and hepatitis C virus RNA, hepatitis C virus genotype, total necroinflammatory activity score, fibrosis score, interferon preparation, and interferon dose.

‡ CI: confidence interval.

§ HCV: hepatitis C virus.

¶ 3-MU group: interferon-α 3 million units thrice weekly for 24 weeks; 6-MU group: interferon-α 6 million units thrice weekly for 24 weeks.

### Dose effect on SVR according to virological risk factors

Since HCV genotype and baseline viral loads were important predictors for SVR to IFN-α, we divided 200 patients into three virological unfavorable risk groups according to the infected HCV genotype and pretreatment HCV RNA levels[14]: low-risk group, absence of both virological risk factors (genotype 1b and pretreatment HCV RNA levels > 0.65 Meq/mL); medium-risk group, presence of only one virological risk factor; and high-risk group, presence of both two virological risk factors. The 6-MU group had significantly higher ETVR than the 3-MU group in all of the three risk groups (p < 0.01, <0.001, and <0.01 for low-, medium-, and high-risk group, respectively, Table 5). The relapsed rate did not differ between 3-MU and 6-MU groups whatever the risk groups. In medium-risk group, patients treated with 6-MU had significantly higher SVR than those with 3-MU (29.5% vs. 7.7%, p < 0.05). However, SVR did not differ between 3-MU and 6-MU groups both in low- and high-risk groups.

**Table 5:** Dose effect of interferon-alpha in chronic hepatitis C patients Dose effect of interferon-alpha on end-of-treatment virological response, virological relapse, and sustained virological response in chronic hepatitis C patients with low-, medium-, and high- virological risk

	Low-risk patients, n/N (%)†		Medium-risk patients, n/N (%)‡		High-risk patients, n/N (%)§	
			
	3-MU group	6-MU group	3-MU group	6-MU group	3-MU group	6-MU group
End-of-treatment virological response	14/19 (73.7)*	32/32 (100)*	19/39 (48.7)**	50/61 (82.0)**	4/18 (22.2)*	21/31 (67.7)*
Virological relapse	1/14 (7.1)	8/32 (25.0)	16/19 (84.0)	32/50 (64.0)	2/4 (50.0)	17/21 (81.0)
Sustained virological response	13/19 (68.4)	24/32 (75.0)	3/39 (7.7)*	18/61 (29.5)*	2/18 (11.1)	4/31 (12.9)

† Low-risk patients, neither genotype 1b nor pretreatment HCV RNA levels>0.65 Meq/mL.

‡ Medium-risk patients, either genotype 1b or pretreatment HCV RNA levels>0.65 Meq/mL.

§High-risk patients, both genotype 1b and pretreatment HCV RNA levels>0.65 Meq/mL.

*, P < 0.01; **, P < 0.001.

### Paired histological examination

**Figure 1:**
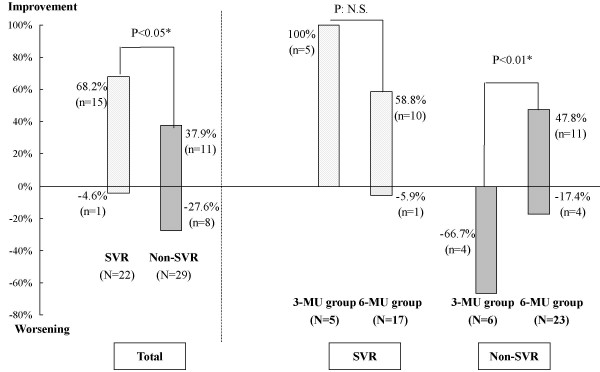
Impact of sustained virological response (SVR) to interferon-alpha therapy on necroinflammatory activity of liver histology. Impact of SVR to interferon-alpha therapy (left side) and of interferon regimens (3-MU *versus*. 6-MU groups, middle and right side) on necroinflammatory activity progression between paired liver histology.

A follow-up liver biopsy was performed 0.5–5.5 years (mean ± SD, 1.9 ± 1.4 years) after end of treatment in 51 patients. A histological improvement with a significant decrease in the mean scores for all necroinflammatory activity (periportal, intralobular, portal inflammation and total necroinflammatory score), but not for fibrosis was observed in the follow-up biopsy (Table 6). The necroinflammatory activity improved in 68.2%, remained stable in 27.3%, and worsened in 4.6% for patients achieved a SVR, in contrast to 37.9%, 34.5%, and 27.6%, respectively, for those with non-SVR (P < 0.05, Fig. 1). Among patients achieving a SVR, there was no difference in histological response between 3-MU and 6-MU groups. Among patients without SVR, there was less frequently worsening of necroinflammatory activity (17.4%) and more activity improvement (47.8%) in 6-MU group, when compared with 3-MU group (66.7% and 0%, respectively, P < 0.01).

**Table 6:** Mean liver histological scores before and after interferon-alpha treatment in 51 Taiwanese chronic hepatitis C patients

	Before treatment	Follow-up†	P value‡
Periportal inflammation	1.25 ± 1.47	0.84 ± 1.27	0.0419
Intralobular necrosis	1.02 ± 1.24	0.49 ± 0.92	0.0129
Portal inflammation	1.88 ± 1.24	1.16 ± 1.32	0.0030
Total necroinflammatory score	4.08 ± 2.86	2.51 ± 3.06	0.0037
Fibrosis	1.12 ± 1.09	1.10 ± 1.22	0.8485

† Mean follow-up time was 1.9 year (range 0.5–5.5 years) after end of treatment.

‡ Wilcoxon's signed rank test.

### Long-term follow-up for viral status and the development of LC and HCC

During the follow-up period, reappearance of serum HCV RNA was found in one patient, who has achieved SVR in 6-MU group, at the 57th month. The durability of SVR was 100% and 97.8% for 3-MU and 6MU group, respectively, in a mean follow-up period of 6.81 years (5.25–9.18 years). The SVR remained similar among three IFN preparations at the end of the 10 years.

Of 136 non-responders in the present study, 50 received a second course of IFN-based treatment. Three of 11 (27.3%) with IFN-α monotherapy and 20 of 39 (51.3%) with IFN-α/ribavirin combination therapy achieved a second SVR. Clinical evaluation, liver biochemistry, α-fetoprotein, and abdominal sonography were performed every 3–6 months for patients without baseline cirrhosis, and every 2–3 months for those with baseline cirrhosis during the follow-up period.

**Figure 2:**
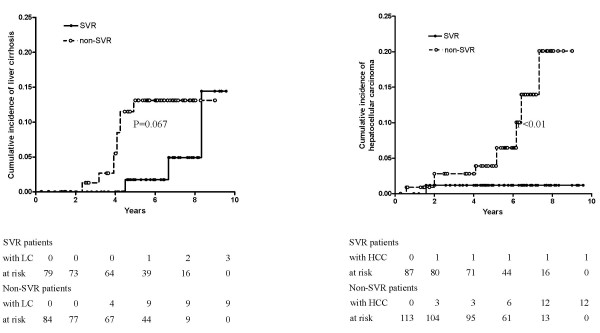
Cumulative incidence of liver cirrhosis (LC) and hepatocellular carcinoma (HCC) among patients treated with interferon-alpha. Left side, cumulative incidence of LC among 163 baseline non-cirrhotic patients with sustained virological response (SVR, solid line) and without SVR (dotted line) to interferon-alpha therapy. P = 0.067 by the log-rank test. The number of LC events and patients at risk at each time point are shown below the graph. Right side, cumulative incidence of HCC among 200 patients with SVR (solid line) and without SVR (dotted line) to interferon-alpha therapy. P < 0.01 by the log-rank test. The number of HCC events and patients at risk at each time point are shown below the graph.

For 163 baseline non-cirrhotic patients, 9 of 84 (10.7%) non-responders and 3 of 79 (3.8%) sustained responders progressed to cirrhosis during a mean follow-up period of 5.52 and 5.74 years, respectively. The difference was borderline significant by using Kaplan-Meier survival analysis (p = 0.067, figure 2, left side). For all 200 patients, HCC was detected in 12 of 113 (10.6%) non-responders and one of 87 (1.1%) sustained responders during a mean follow-up period of 5.67 and 5.73 years, respectively. The difference was significant by using Kaplan-Meier survival analysis (p < 0.01, figure 2, right side).

## Discussion

In the present study, we have investigated the dose effect of IFN-α therapy on a group of Taiwanese patients with chronic hepatitis C in a 10-year follow-up. Our results demonstrated that 6 MU IFN-α, thrice weekly for 24 weeks, had better efficacy (2.3-fold) than 3 MU, thrice weekly for 24 weeks, in both virological and histological responses. Improved efficacy was mainly seen in subgroups of patients with one of the two unfavorable virologic factors, the medium-risk group, in the current study. The 6-MU regimen was as tolerable as standard 3-MU regimen for Taiwanese patients. Both regimens had good durability of SVR.

Treatment with IFN-α was the first approved therapy but a SVR could be achieved in only 8–20% of chronic hepatitis C patients treated with a standard regimen of IFN-α monotherapy (3 MU thrice weekly for 24 weeks) [3-8]. Therefore, how to improve the efficacy of IFN-α therapy is the concern of many studies. Until recently, 48 weeks of IFN-α, 3 MU thrice weekly was recommended for the treatment of chronic hepatitis C[5]. Concerning the suffering from long-term IFN therapy, we conducted higher dose of IFN regimen, 6 MU thrice weekly for 24 weeks, which total dosage was equal to that of 3 MU, thrice weekly for 48 weeks. In the present study, 6-MU regimen was observed to achieve significantly higher rates of ETVR and SVR than 3-MU regimen, but had no benefit on reducing the relapse rate. In contrast to the duration effect on improvement of SVR by reducing the relapse rate after end-of-treatment, our results implicated the dose effect on the initial response of HCV to IFN therapy[5,7,8]. High dose regimen as well as induction therapy could result in early clearance of HCV viremia, which is very important in the prediction of IFN response[10,15,16].

In the present study, the incidence of discontinuation and most of the major adverse events were similar between standard dose and higher dose of IFN therapy, despite that the higher dose of IFN regimen had significantly higher incidence of psychological manifestations. However, the psychological manifestations, mainly presented as irritability, could be controlled well with minor tranquillizers.

Consistent with previous reports[2,3,9,10,14], we confirmed the associations of pretreatment serum HCV RNA levels, HCV genotype 1b and presence of cirrhosis with response to IFN-α treatment in chronic hepatitis C patients by using stepwise logistic regression model. According to previous studies for Taiwanese patients, we divided our patients into three virological risk groups based on the presence/absence of HCV genotype 1b and the baseline viral loads greater than the cut-off point, 0.65 Meq/mL, or not[14]. As observed in the present study, 6-MU regimen could achieve significantly higher rate of ETVR but could not decrease the relapse rate in all of the three risk groups. Although our logistic regression model predicted a 2.3-times increase in probability for a SVR to occur in the 6-MU regimen versus the 3-MU regimen, the significantly better efficacy of 6-MU regimen on SVR was only observed in patients with one virological risk factor. Therefore, how to enhance the ETVR for the medium- and high-risk groups and to reduce the relapse rates for all of the three risk groups will be the key to improve SVR. Extended therapy and/or combination with ribavirin might be considered to overcome the unfavorable virological risk factors[16,17]. Since the unfavorable virological risk factors are unchangeable, tailored regimens of IFN with or without ribavirin combination therapy according to the virological status are the only way to improve the efficiency of HCV eradication, as recommended in recent consensus on management of chronic hepatitis C[18,19].

In accordance with previous studies [20-22], a significant decrease in necroinflammatory activity of liver histology was observed in the current study. However, our material was unable to show whether the IFN treatment resulted in cessation of fibrogenesis, regardless of IFN regimen and virological response (data not shown). Indeed, no firm conclusion can be drawn about whether the fibrosis remaining at long-term follow-up was irreversible and established or whether it will diminish with even longer follow-up[22]. Further analysis of histological response, stratified by IFN regimen and SVR, we found that 6-MU regimen could achieve histological improvement not only in SVR but also in non-SVR. By contrast, most of non-SVR treated with 3-MU regimen had histological worsening. These results presumed the prominent benefit of high dose IFN on the treatment of chronic hepatitis C.

Similar to other reports[23,24], the long-term prognosis was excellent in this well-defined material of SVR. Serum HCV RNA was persistently undetectable in 63 of 64 patients with SVR. The only one patient, who had reappearance of serum HCV RNA concomitant with abnormal ALT level at the 57th month of follow-up, is a resident of an HCV hyperendemic area, Tzukuan[25]. Whether this is reinfection[26] or recurrence[23,24] of HCV remains to be clarified by phylogenetic analysis of viral genome. Also our results confirm that IFN therapy, when associated with response, reduces the incidence of LC and HCC among chronic hepatitis C patients[27].

A greater efficacy in treatment of chronic hepatitis C was observed in combination with IFN-α and ribavirin[24], which has been available in Taiwan since August 1998 and becomes to be the recommendations for chronic hepatitis C patients[18,19]. More recently, combination of pegylated IFN-α plus ribavirin shows more effective and convenient and may replace the current standard of IFN-α plus ribavirin[28,29]. The results of current study could provide decision-making information for future therapeutic strategies of individualizing dose and duration of standard or pegylated IFN-α treatment in combination with ribavirin according to the baseline virological predictors to improve the efficiency of HCV eradication. However, pegylated IFN-α and ribavirin combination therapy is expensive and might carry potential side effects. The present higher dose regimen of IFN monotherapy might be suggested for patients who are contraindicated to ribavirin and/or pegylated IFN-α therapy[30].

## Conclusion

We confirm the dose effect of IFN in chronic hepatitis C. Six-MU regimen had better efficacy than standard 3-MU regimen in both of virological and histological responses, and was as tolerable as 3-MU regimen. Nearly all sustained virological responders had a durable response and the sustained response had a preventive effect on the development of HCV-associated LC and HCC. Since the unfavorable predictors, high pretreatment HCV RNA levels, infected with genotype 1b, and presence of cirrhosis, are unchangeable, adjustment of IFN dose and duration according to the unfavorable factors is important to achieve a better efficacy/risk ratio. Our results could provide decision-making information for future therapeutic strategies of individualizing dose and duration of standard or pegylated IFN-α treatment in combination with ribavirin according to baseline predictors.

## List of abbreviations

Hepatitis C virus: HCV; interferon-alpha: IFN-α; million units: MU; liver cirrhosis: LC, hepatocellular carcinoma: HCC, end-of-treatment virologic responder: ETVR, sustained virologic responder: SVR; reverse transcription polymerase chain reaction assay: RT-PCR; million equivalent: Meq.

## Competing interests

The author(s) declare that they have no competing interests.

## Authors' contributions

MLY carried out the laboratory work and molecular virologic studies, participated in the collection and clinical evaluation of patients, performed the statistical analysis, and drafted the manuscript. CYD, SCC and LPL participated in the laboratory work and molecular virologic studies, and in the collection and clinical evaluation of patients. MYH, ZYL, MYH, JFT and LYW, participated in the collection and clinical evaluation of patients. WYC participated in the design of the study. WLC conceived of the study, and participated in its design and coordination. All authors read and approved the final manuscript.

## Pre-publication history

The pre-publication history for this paper can be accessed here:


